# Supplementary material for: Regulation of gene expression and RNA editing in Drosophila adapting to divergent microclimates
Source: Nat Commun. 2017 Nov 17;8:1570. doi: 10.1038/s41467-017-01658-2 (PMC5691062; doi:10.1038/s41467-017-01658-2)
Supplement: Supplementary file 1 — Supplementary Information [file 41467_2017_1658_MOESM1_ESM.pdf]

Supplementary Table 1: Annotation and gene information for editing sites which are differentially edited between NFS1 and SFS

Gene	Molecular Function	Editing Site	Annotation	Dataset
prominin	protein binding	chr2R:20306770	Nonsyn, Asn->Ser	mmPCR-seq, RNA-seq
		chr2R:20306773	Nonsyn, Tyr->Cys	mmPCR-seq
Dynein heavy chain at 93AB	ATPase	chr3R:16861033	Nonsyn, Gln->Arg	mmPCR-seq
		chr3R:16860925	Nonsyn, Gln->Arg	mmPCR-seq
		chr3R:16859660	Nonsyn, Ile->Val	mmPCR-seq
		chr3R:16854033	Nonsyn, Ile->Val	mmPCR-seq
CG42492	N/A	chrX:5524375	Nonsyn, Tyr->Cys	mmPCR-seq
falafel	protein binding	chr3R:9518315	3'UTR	mmPCR-seq
		chr3R:9518314	3'UTR	mmPCR-seq
CG14142	N/A	chr3L:11168895	Nonsyn, Gln->Arg	mmPCR-seq
		chr3L:11168876	Nonsyn, Thr->Ala	mmPCR-seq
Ryanodine receptor	ryanodine-sensitive calcium-release channel	chr2R:4772736	Nonsyn, Lys->Arg	mmPCR-seq
		chr2R:4772882	Nonsyn, Ser->Gly	mmPCR-seq
CG31462	Immunoglobulin	chr3R:4212460	Nonsyn, Asn->Asp	mmPCR-seq
		chr3R:4212480	Nonsyn, Lys->Arg	mmPCR-seq
NMDA receptor 1	NMDA glutamate receptor	chr3R:1337783	Nonsyn, Met->Val	mmPCR-seq
Ca2+-channel protein α1 subunit T	Voltage-gated calcium channel	chrX:6003904	Nonsyn, Thr->Ala	mmPCR-seq
		chrX:6009458	Nonsyn, Ser->Gly	mmPCR-seq
CG42540	N/A	chr3L:4590708	3'UTR	mmPCR-seq
CG31935	GTPase Activator	chr2L:1609982	Nonsyn, Ser->Gly	mmPCR-seq
Diacyl glycerol kinase ε	Diacyl glycerol kinase	chr2R:8646087	Nonsyn, Ile->Met	mmPCR-seq
Octopamine β3 receptor	Octopamine receptor	chr3R:8343182	Nonsyn, Lys->Arg	mmPCR-seq
		chr3R:8343173	Nonsyn, Lys->Arg	mmPCR-seq
Shaker	Voltage-gated potassium channel	chrX:17832044	Intronic	RNA-seq
G protein subunit γ at 30A	protein heterodimerization	chr2L:9280671	Intronic	RNA-seq
CG13739	protein dimerization	chr2R:5231745	Intronic	RNA-seq
VAMP-associated protein 33kDa	FFAT motif binding	chrX:3847324	3'UTR	RNA-seq
short stop	protein binding	chr2R:9781085	Nonsyn, Thr->Ala	RNA-seq
Na+/H+ hydrogen exchanger 3	sodium:proton antiporter	chr2L:6682304	Nonsyn, Ser->Gly	RNA-seq
Intergenic	N/A	chr2L:7643369	Intergenic	RNA-seq
		chrX:14721060	Intergenic	RNA-seq

The sites represented have an FDR-adjusted p-value < 0.05 and an editing difference of at least 5% between the NFS1 and SFS fly populations.

Supplementary Table 2: Annotation and gene information for sites which are differentially edited between NFS2 and SFS

Gene	Molecular Function	Editing Site	Annotation	Dataset
CG14142	N/A	chr3L:11168868	Nonsyn, Glu->Gly	mmPCR-seq
		chr3L:11168876	Nonsyn, Thr->Ala	mmPCR-seq
		chr3L:11168895	Nonsyn, Gln->Arg	mmPCR-seq
CG14408	protein kinase inhibitor	chrX:14723854	Nonsyn, Lys->Glu	mmPCR-seq
uncoordinated 79	N/A	chr3R:15056819	Nonsyn, Asn->Asp	mmPCR-seq
		chr3R:15064567	Nonsyn, Glu->Gly	mmPCR-seq
straightjacket	voltage-gated calcium channel	chr2R:9697052	Nonsyn, Ile->Val	mmPCR-seq
CG34355	N/A	chr3R:19674259	Nonsyn, Lys->Glu	mmPCR-seq
Rad, Gem/Kir family member 1	GTPase	chr2R:15213814	Nonsyn, Gln->Arg	mmPCR-seq
CG34357	guanylate cyclase	chr3R:319616	Nonsyn, Gln->Arg	mmPCR-seq
CG10185	N/A	chr3R:11891689	Nonsyn, Ile->Met	mmPCR-seq
CG42795	GTPase activator	chr3R:6071147	Nonsyn, Ser->Gly	mmPCR-seq
CG42540	N/A	chr3L:4590708	3'UTR	mmPCR-seq
Endophilin A	lysophosphatidic acid acyltransferase	chr3R:14735875	3'UTR	RNA-seq
Major Facilitator Superfamily Transporter 17	transmembrane transporter	chr2RHet:1267431	Intronic	RNA-seq
		chr2RHet:1269010	Intronic	RNA-seq
retinal degeneration A	diacylglycerol kinase	chrX:8818718	Intronic	RNA-seq
		chrX:8818719	Intronic	RNA-seq
CG42492	N/A	chrX:5524246	Nonsyn, Asp->Gly	RNA-seq
Na/Ca-exchange protein	calcium:sodium antiporter	chr3R:16819332	Nonsyn, Asp->Gly	RNA-seq
retinophilin	N/A	chr3R:1062051	3'UTR	RNA-seq
kuzbanian	metalloendopeptidase	chr2L:13550505	Intronic	RNA-seq
Dopamine/Ecdysteroid receptor	G-protein coupled amine receptor	chr3L:4369459	Nonsyn, Asn->Ser	RNA-seq
Cysteine string protein	heat shock protein 40/DnaJ co-chaperone	chr3L:22266244	3'UTR	RNA-seq

The sites represented have an FDR-adjusted p-value < 0.05 and an editing difference of at least 5% between the NFS2 and SFS fly populations.

Supplementary Table 3: The 10 most differentiated SNPs in *prominin*

SNP	Allele frequency in NFS1	Allele frequency in SFS
chr2R:20304899	0.933333	0
chr2R:20305901	0.067708	0.86351
chr2R:20306664	0.888889	0.165644
chr2R:20307327	0.914573	0
chr2R:20307717	0.890909	0
chr2R:20309987	0.870229	0
chr2R:20312645	0	0.725309
chr2R:20312847	1	0.26799
chr2R:20313061	0.857798	0
chr2R:20314480	0.090517	0.990099

The SNPs are sorted by position, and the SNP highlighted in blue is in *prominin*'s predicted ECS.

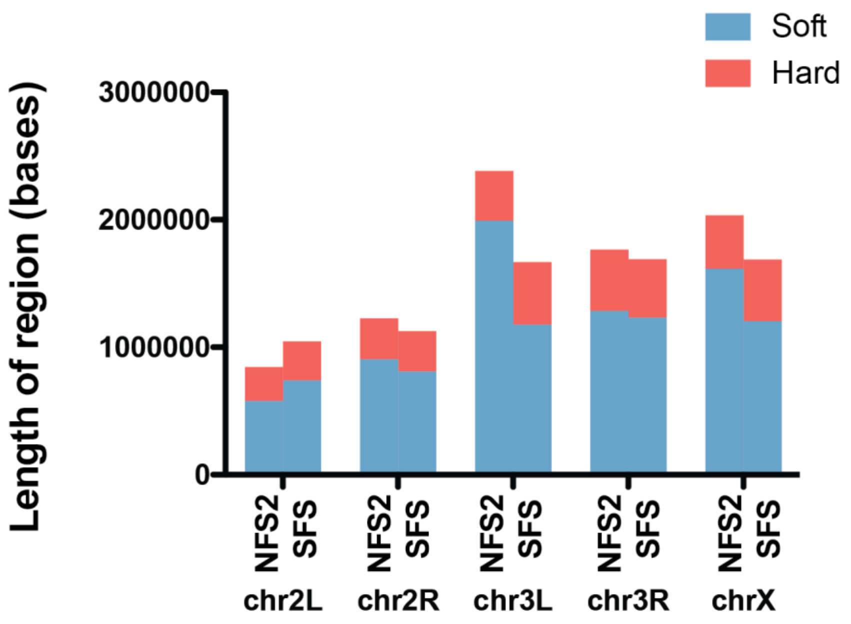

Supplementary Figure 1: Comparing the sizes of selective sweeps between the NFS2 and SFS fly populations. Bar plot showing the length of hard sweep regions (red) and soft sweep regions (blue) in 2 NFS2 and 12 SFS lines for different chromosomes.

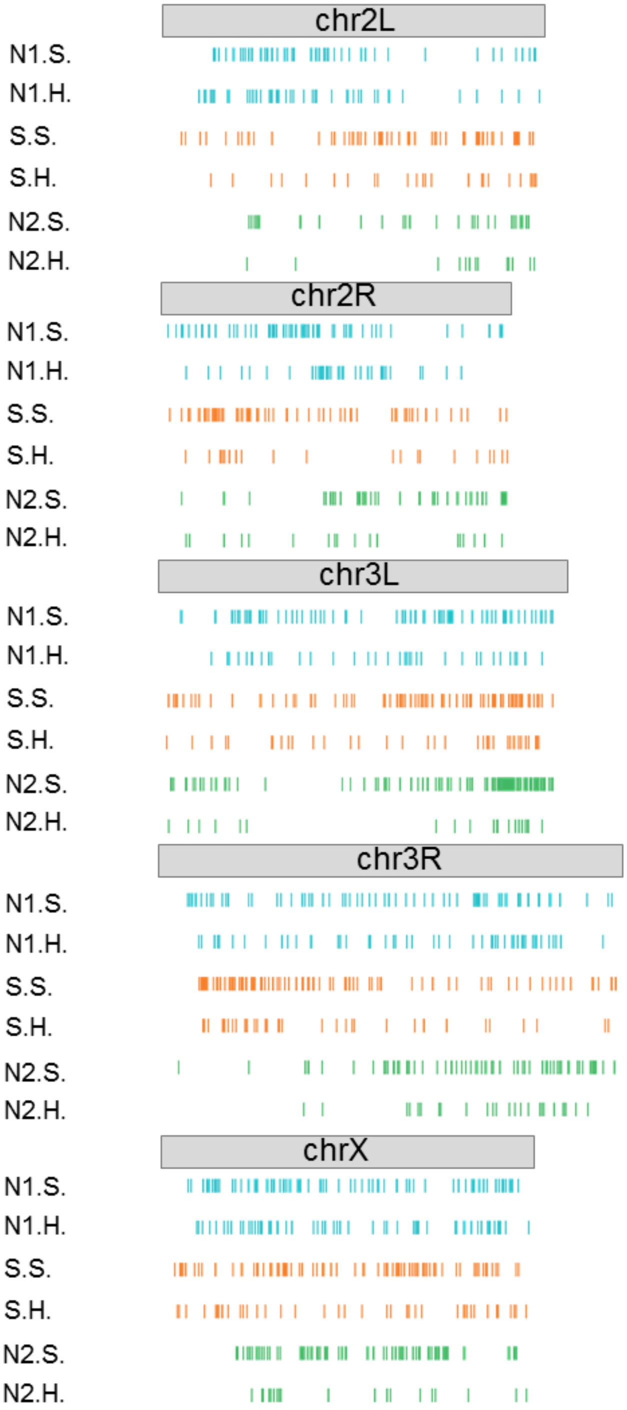

Supplementary Figure 2: Comparing the locations of selective sweeps between the NFS1, SFS, and NFS2 fly populations. Diagram showing the location of selective sweeps across different chromosomes for the NFS1, SFS, and NFS2 fly populations, represented by 4, 12, and 2 fly lines, respectively. "N1.S." represents NFS1 soft sweeps, "N1.H." represents NFS1 hard sweeps, "S.S." represents SFS soft sweeps, "S.H." represents SFS hard sweeps, "N2.S." represents NFS2 soft sweeps, and "N2.H." represents NFS2 hard sweeps.

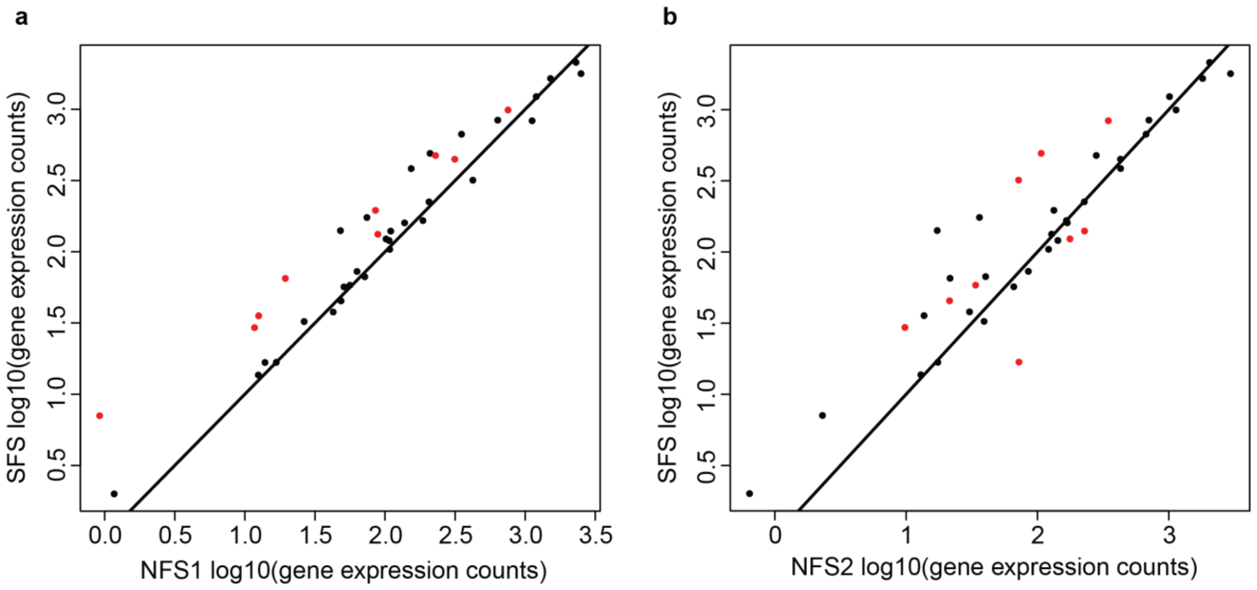

Supplementary Figure 3: Comparing Glutathione S-transferase gene expression between different Evolution Canyon fly populations. Scatterplot comparing Glutathione S-transferase gene expression in whole bodies between the 8 NFS1 and 16 SFS fly lines (a) and between the 5 NFS2 and 16 SFS fly lines (b). Points in red represent significantly differentially expressed genes (Benjamini-Hochberg adjusted p-value < 0.05, Wald test from DESeq2).

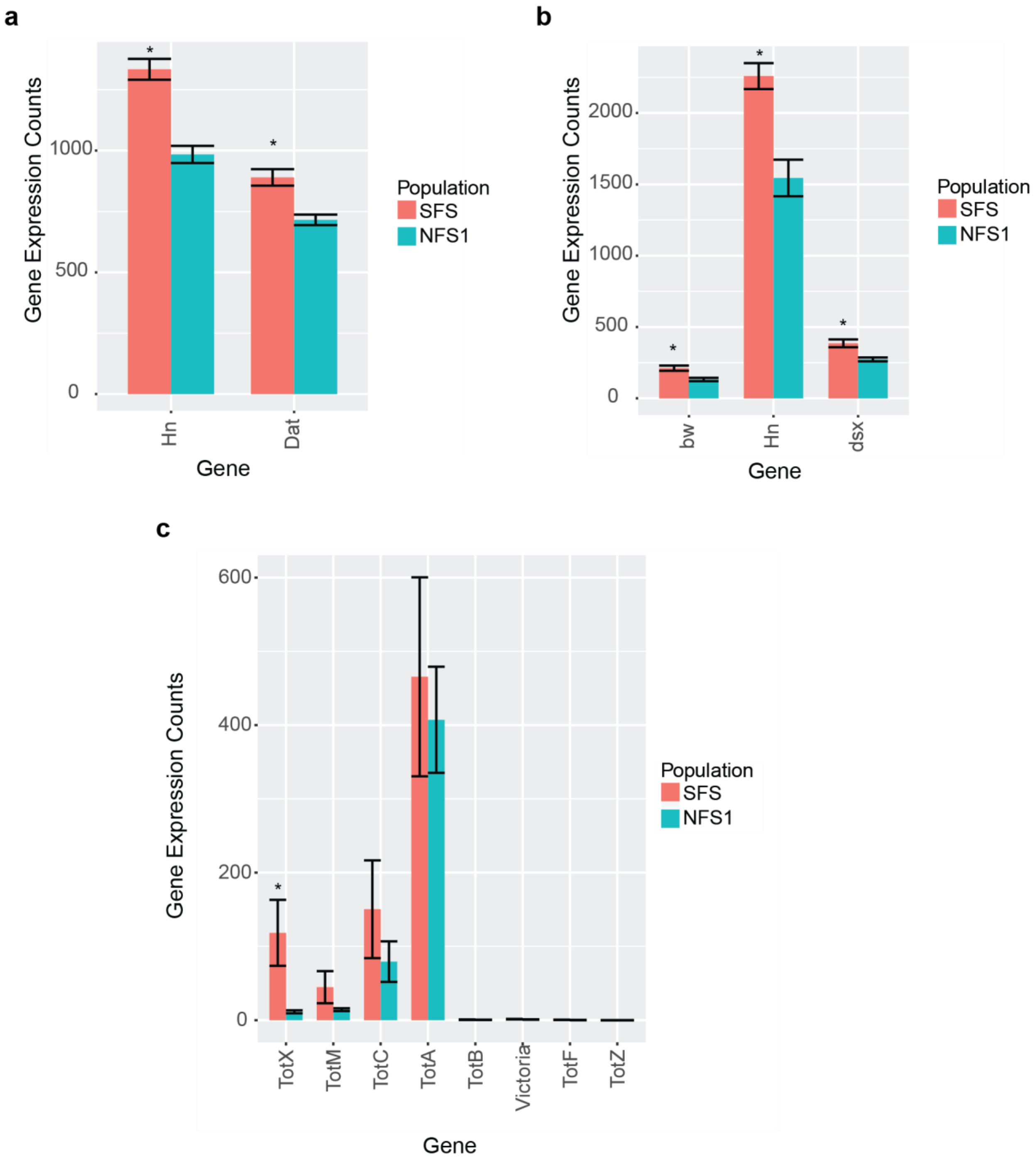

Supplementary Figure 4: Comparing the expression of specific pigmentation and heat response genes between the SFS and NFS1 fly populations. Average gene expression counts from RNA-seq data for (a) Pigmentation genes in whole bodies, (b) Pigmentation genes in heads, and (c) *Turandot* genes in whole bodies in the 16 SFS and 8 NFS1 fly lines. An asterisk represents genes with significantly different expression between SFS and NFS1 (Benjamini-Hochberg adjusted p-value < 0.05, Wald test from DESeq2). Error bars represent standard error of the mean. SFS gene expression is shown in red, while NFS1 gene expression is shown in turquoise.

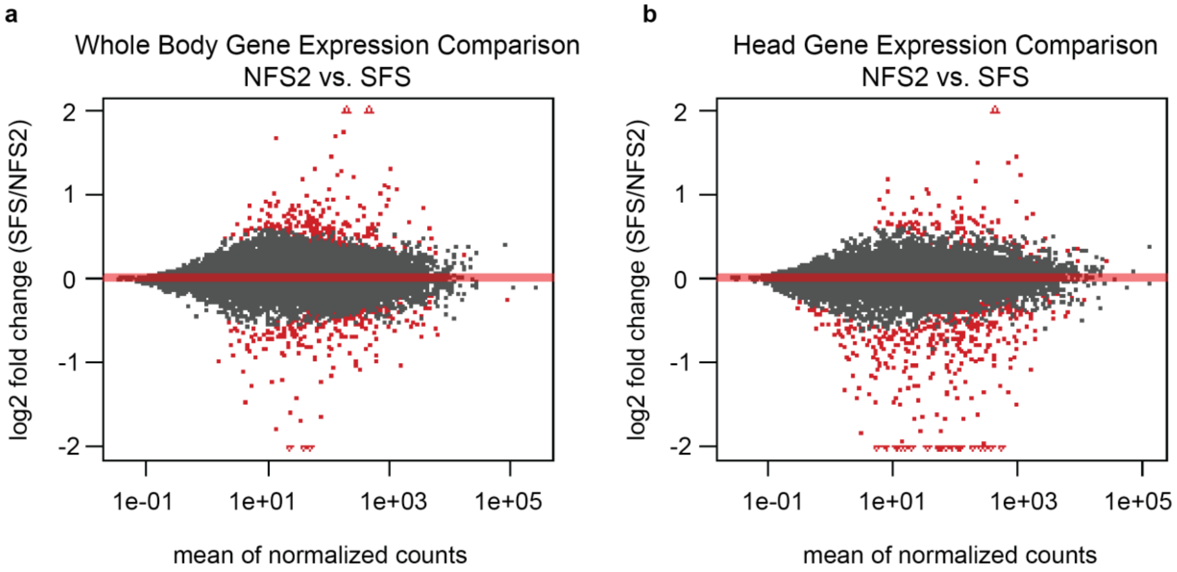

Supplementary Figure 5: Comparing gene expression between the NFS2 and SFS fly populations. Scatter plot showing gene expression differences between the 5 NFS2 and 16 SFS fly lines in whole bodies (a) and heads (b). Red points represent genes with significantly different expression levels (Benjamini-Hochberg adjusted p-value < 0.05, Wald test, DESeq2).

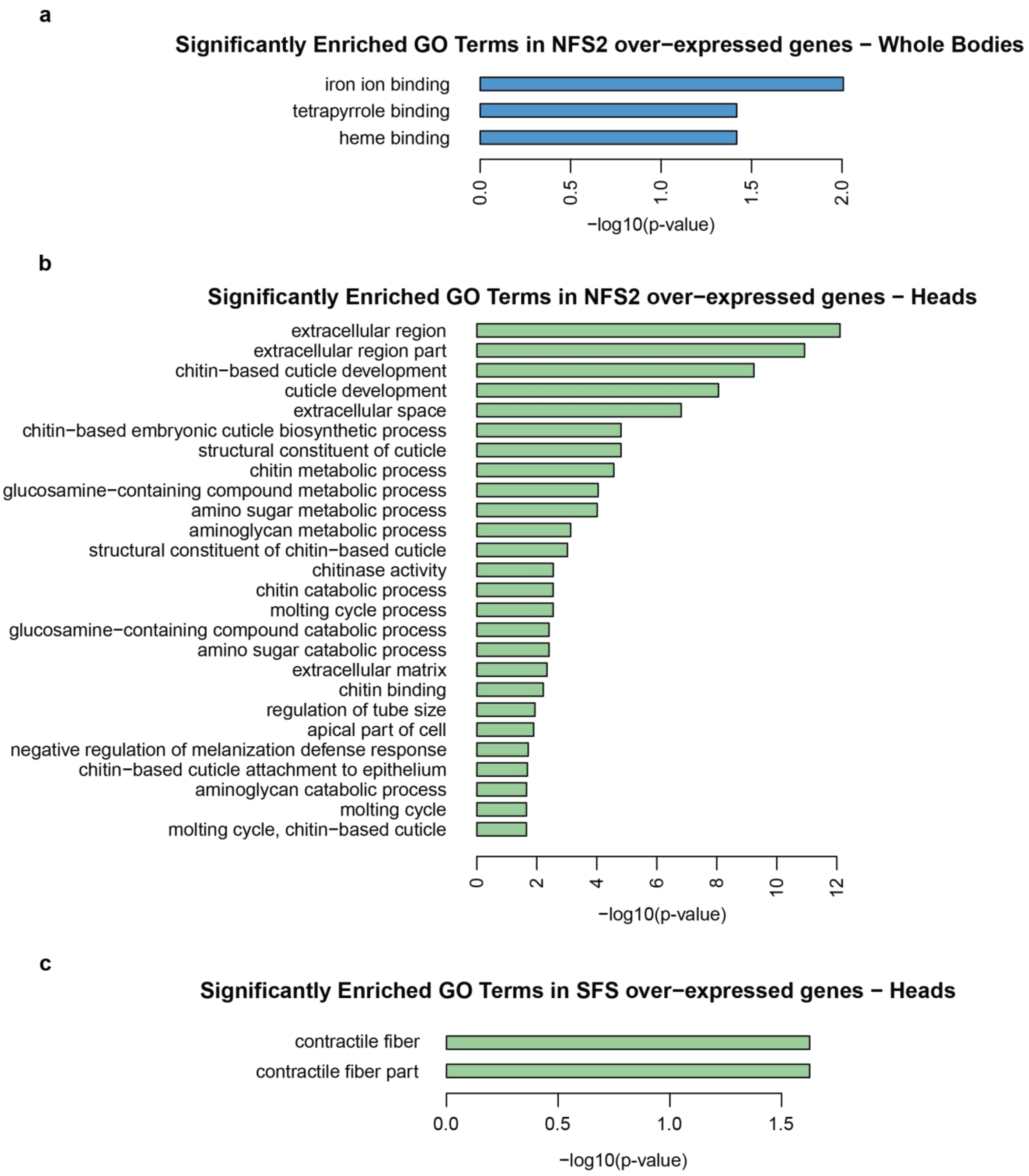

Supplementary Figure 6: GO-term enrichment analysis for differentially expressed genes between the NFS2 and SFS fly populations. Significantly enriched GO-terms and associated p-values of genes over-expressed in NFS2 in whole bodies (a), genes over-expressed in NFS2 in heads (b), and genes over-expressed in SFS in heads (c) (Benjamini-Hochberg adjusted p-value < 0.05, GOseq using the Wallenius approximation).

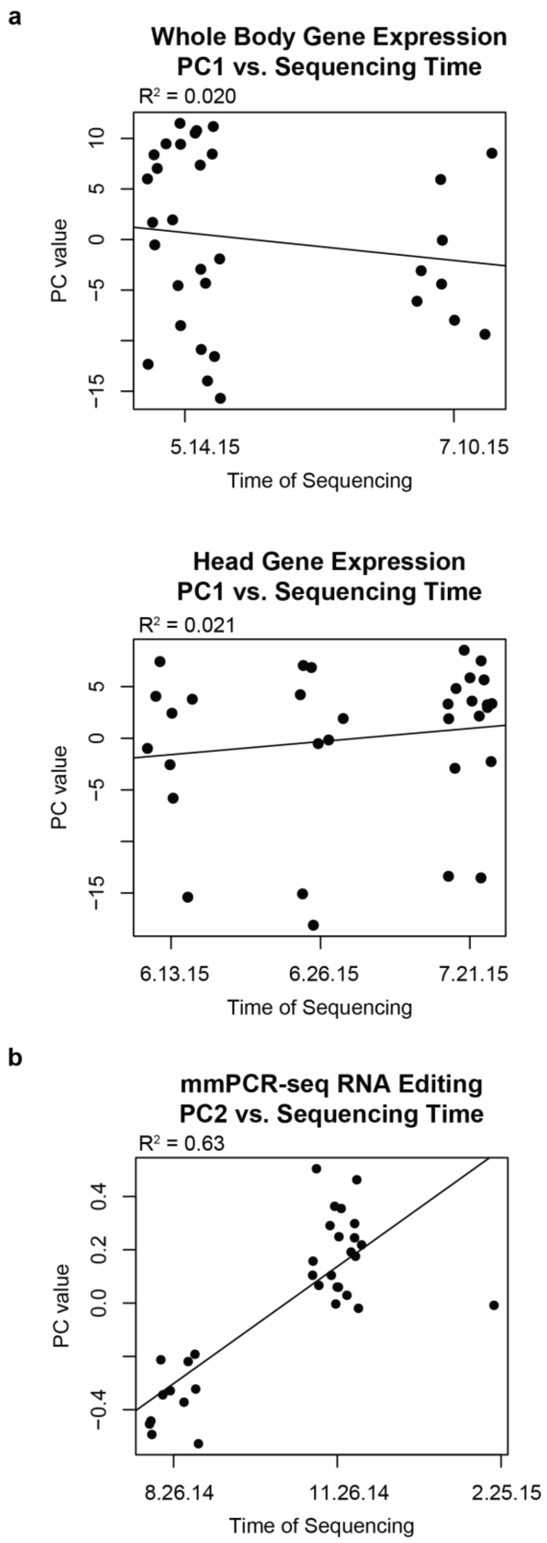

Supplementary Figure 7: Correlations between gene expression and RNA editing PCs (Principal Components) and time of sequencing. Scatterplots comparing PC values with time of sequencing for (a) gene expression and (b) mmPCR-seq RNA editing Principal Component Analyses.

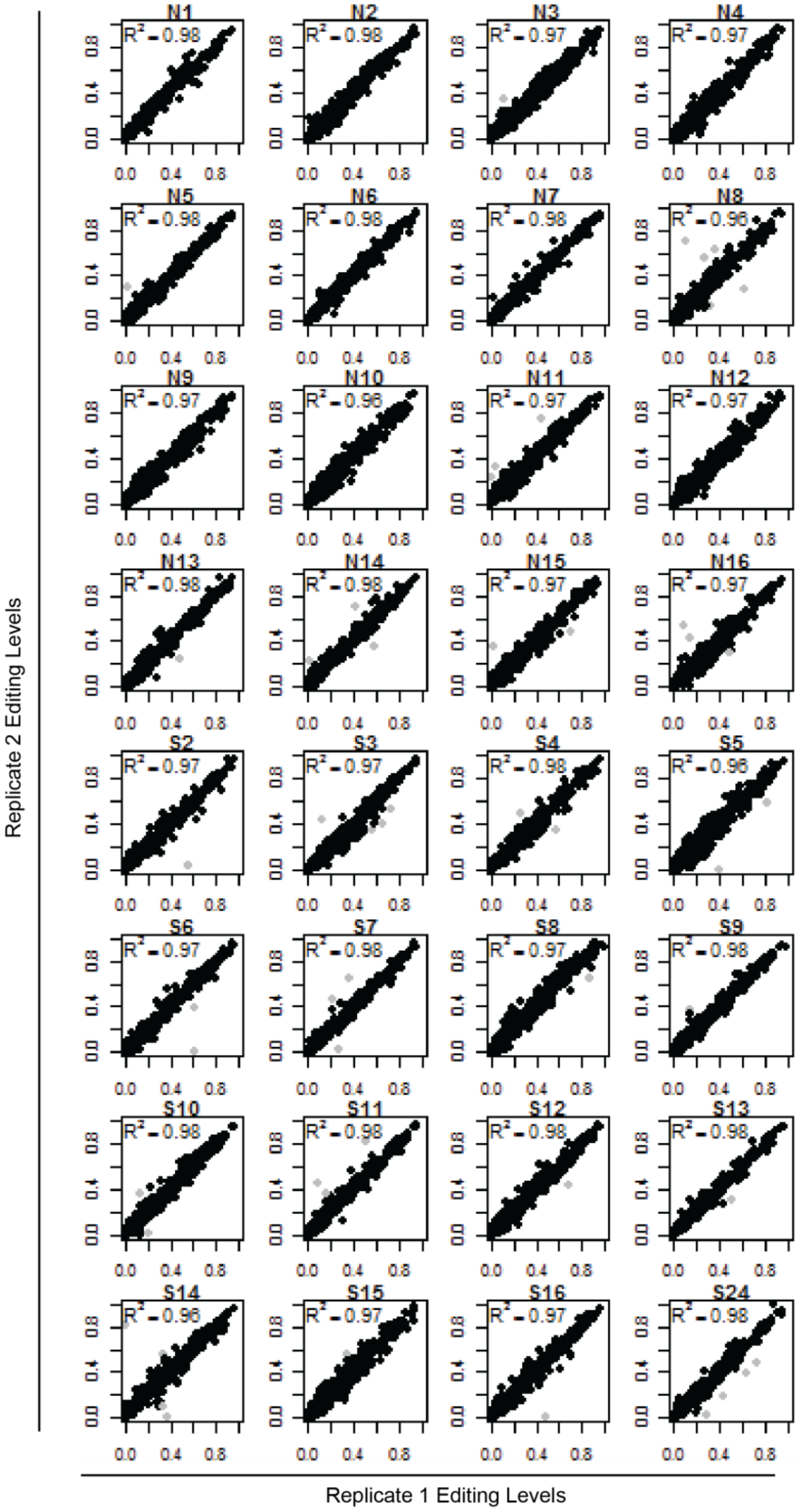

Supplementary Figure 8: Comparing mmPCR-seq editing levels between biological replicates of Evolution Canyon flies raised at 25˚C. Scatterplots comparing mmPCR-seq editing levels of biological replicates (one single fly per replicate) for each of the 32 Evolution Canyon lines raised at 25˚C. Gray points represent sites with ≥ 20% editing level difference between replicates, which were not included in subsequent analyses.

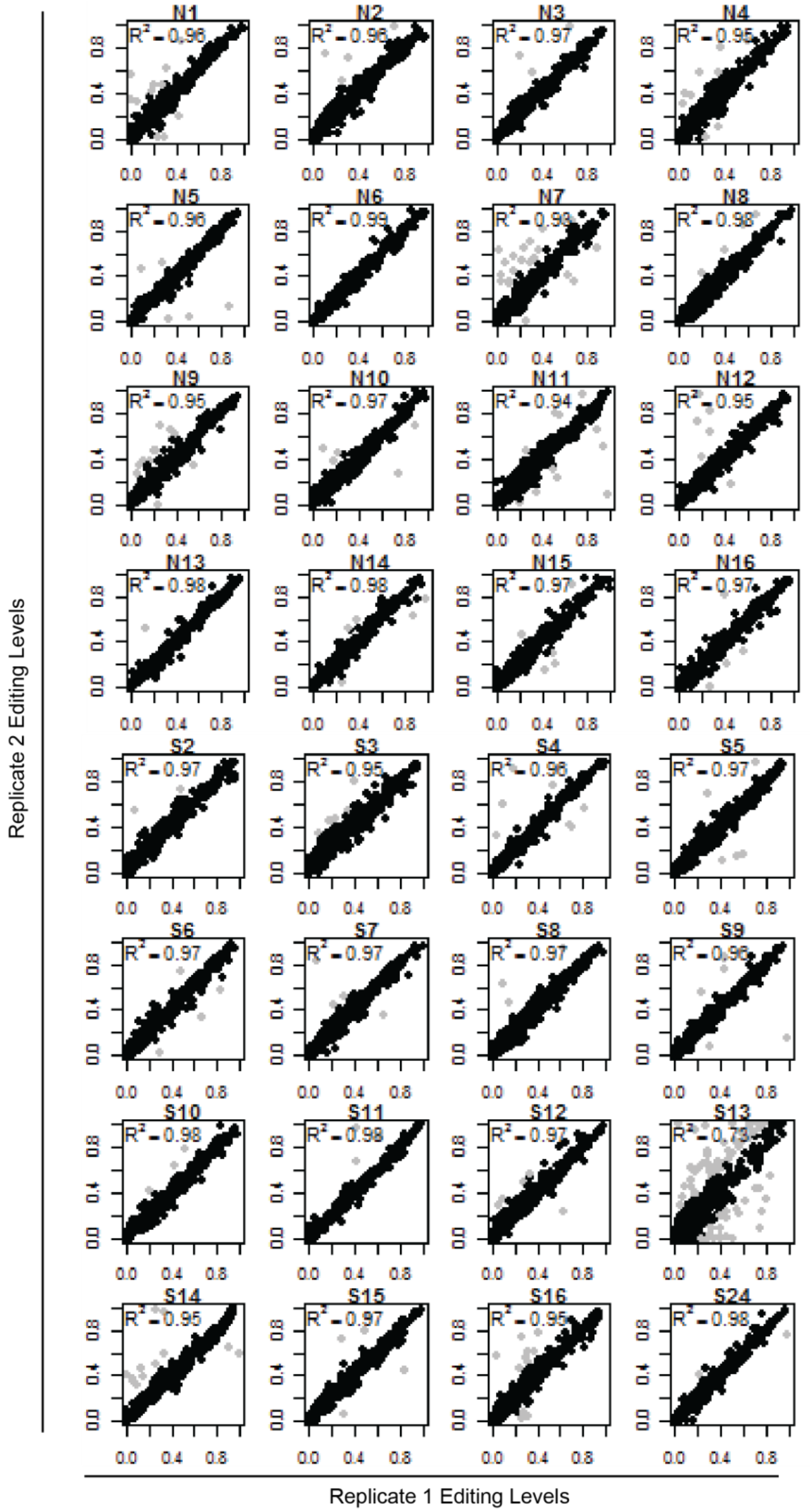

Supplementary Figure 9: Comparing mmPCR-seq editing levels between biological replicates of Evolution Canyon flies raised at 18˚C. Scatterplots comparing mmPCR-seq editing levels of biological replicates (one single fly per replicates) for each of the 32 Evolution Canyon lines raised at 18˚C. Gray points represent sites with ≥ 20% editing level difference between replicates, which were not included in subsequent analyses.

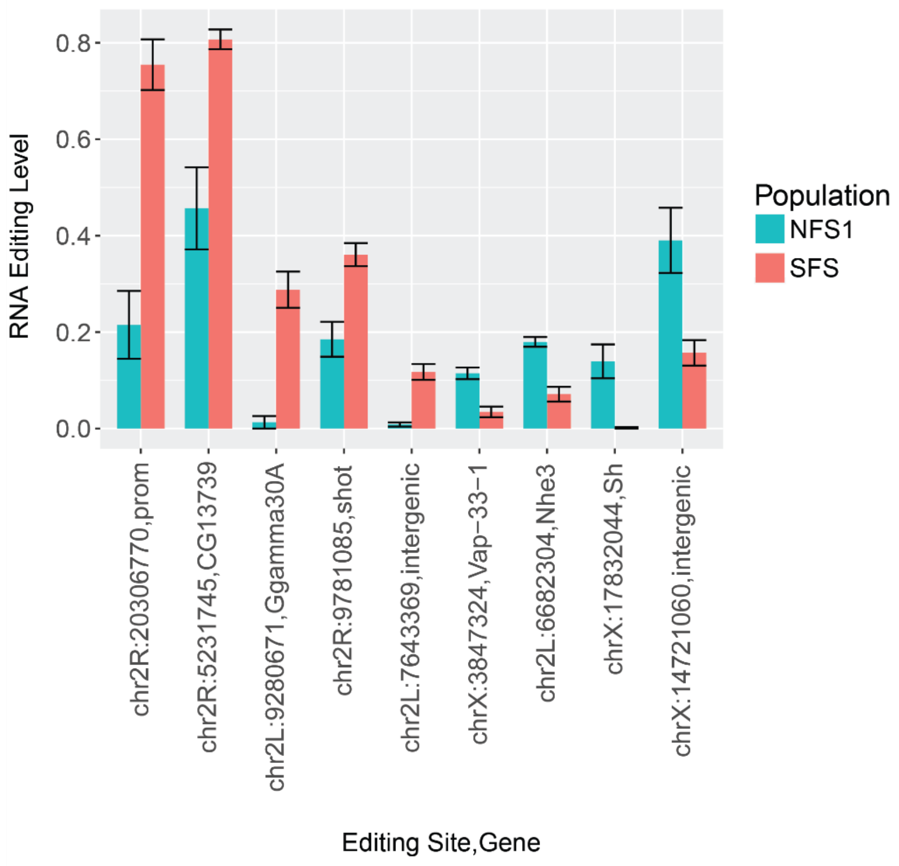

Supplementary Figure 10: Significantly differentially edited sites between NFS1 and SFS flies identified from head RNA-seq data. Barplot showing the average editing levels of the NFS1 (turquoise) and SFS (red) fly lines for significant sites identified between these two populations from head RNA-seq (FDR-adjusted p-value < 0.05). Error bars represent standard error of the mean. The number of fly lines represented for each site per population ranges from 7 to 8 for NFS1 and 14 to 16 for SFS; see Supplementary Data 7.

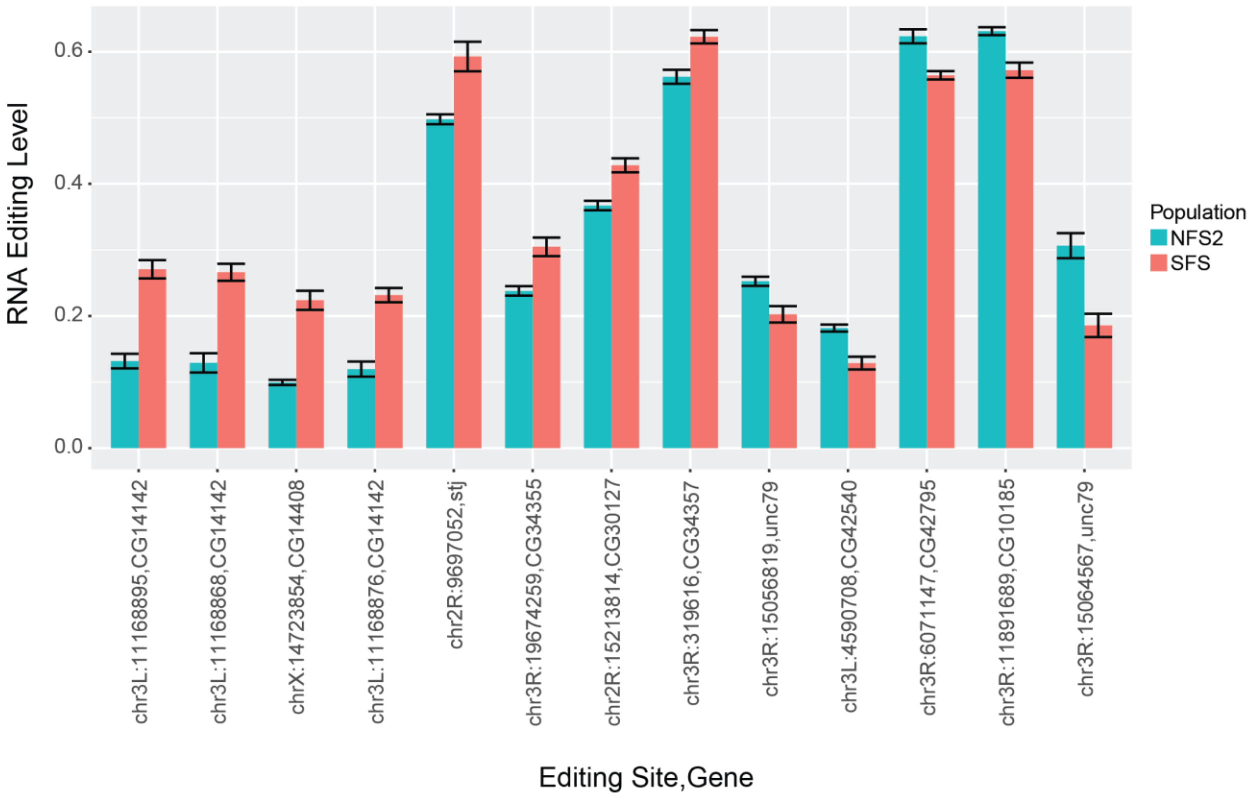

Supplementary Figure 11: Significantly differentially edited sites between NFS2 and SFS flies identified from mmPCR-seq data. Barplot showing the average editing levels of the NFS2 (turquoise) and SFS (red) fly lines for significant sites identified between these two populations from mmPCR-seq (t-test, FDR-adjusted p-value < 0.05, editing level difference $\geq 5\%$). Error bars represent standard error of the mean. The number of fly lines represented for each site per population ranges from 3 to 5 for NFS2 and 12 to 16 for SFS; see Supplementary Data 6.

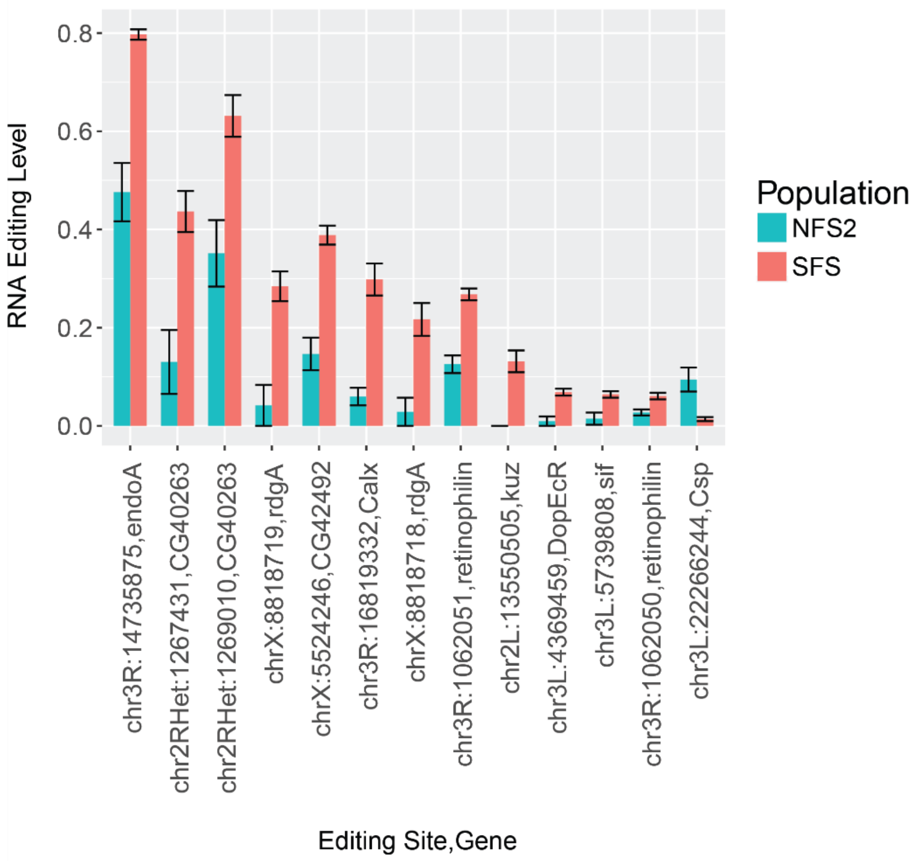

Supplementary Figure 12: Significantly differentially edited sites between NFS2 and SFS flies identified from head RNA-seq data. Barplot showing the average editing levels of the NFS2 (turquoise) and SFS (red) fly lines for significant sites identified between these two populations from head RNA-seq (FDR-adjusted p-value < 0.05). Error bars represent standard error of the mean. The number of fly lines represented for each site per population ranges from 3 to 5 for NFS2 and 14 to 16 for SFS; see Supplementary Data 7.

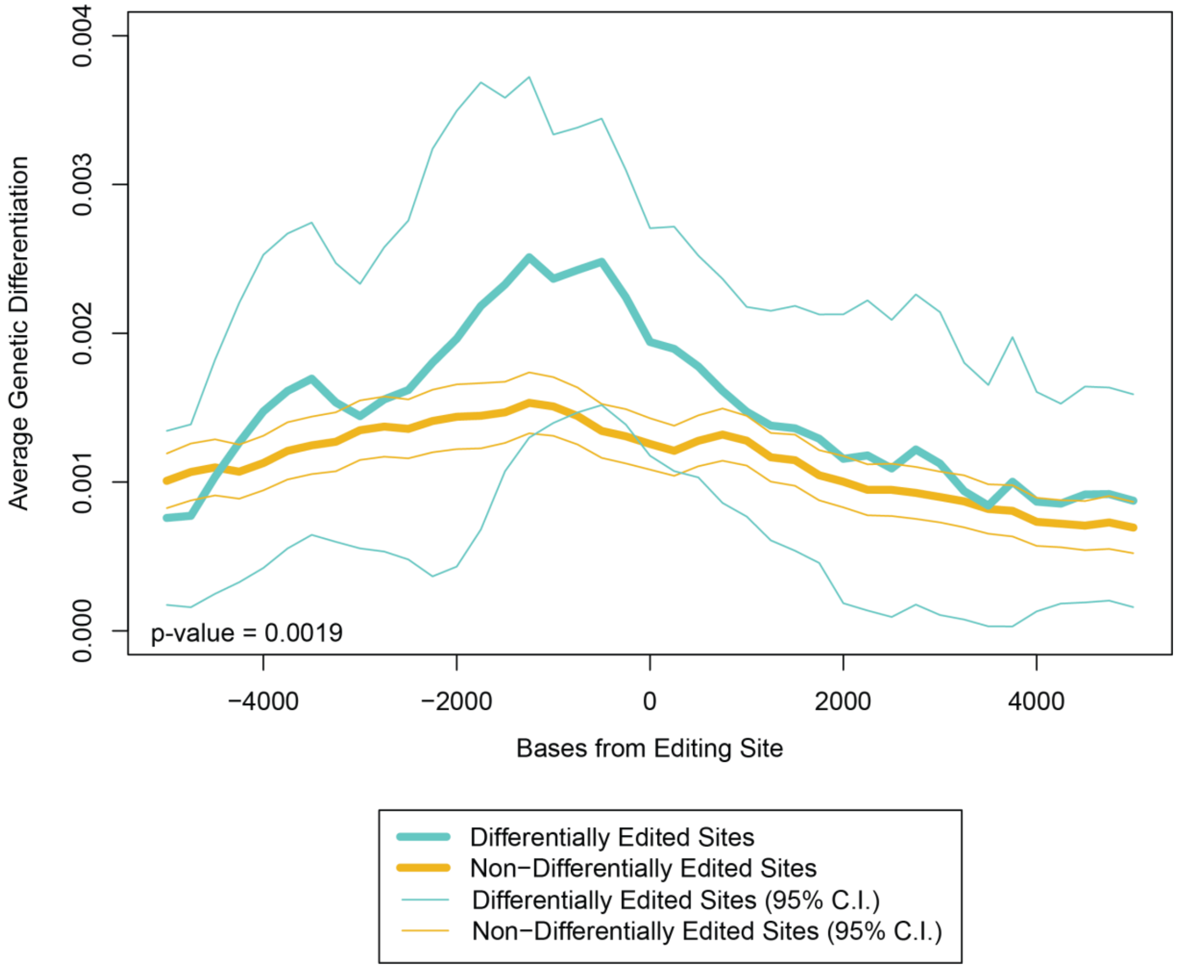

Supplementary Figure 13: Increased genetic differentiation around differentially edited sites between the NFS2 and SFS fly populations. Plot showing significant enrichment of differentiated SNPs near significantly differentially edited sites (turquoise) compared to non-differentially edited sites (gold) between the NFS2 and SFS fly populations, along with the 95% confidence interval (p-value = 0.0019, one-sided KS test).

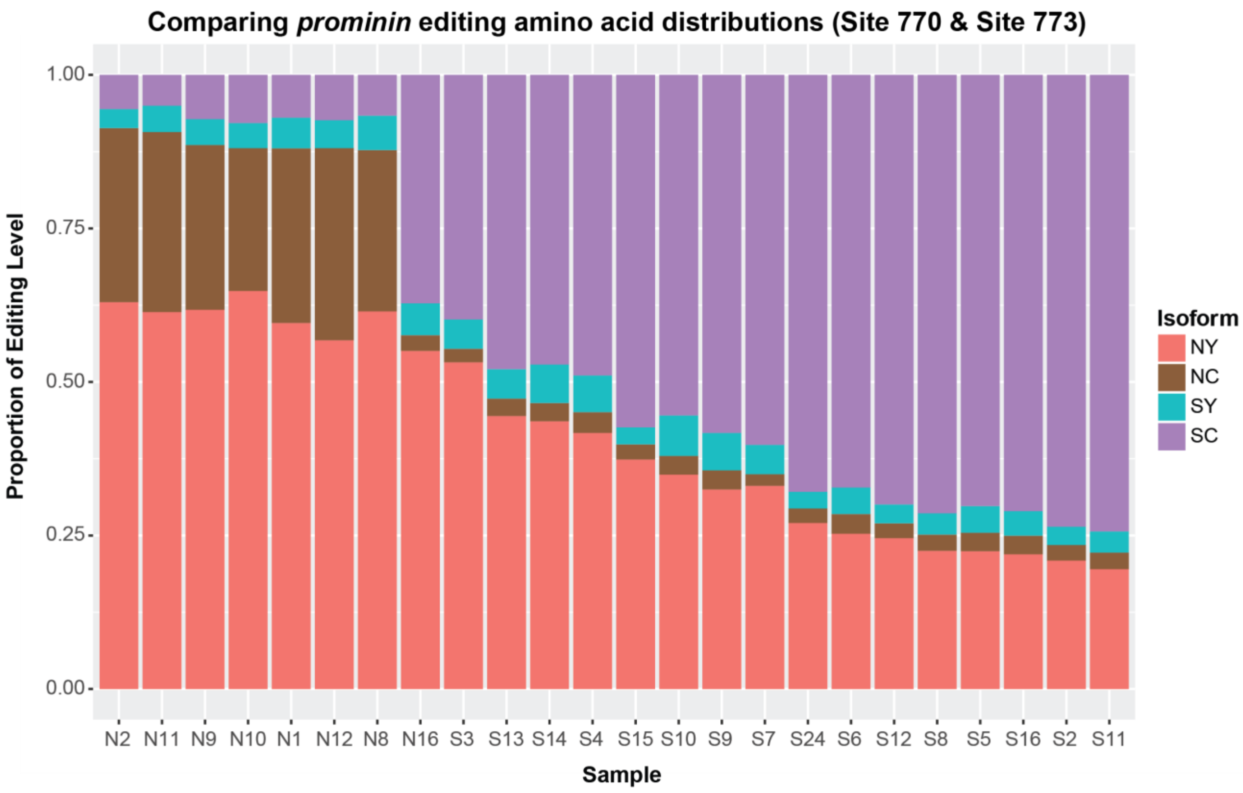

Supplementary Figure 14: Comparing *prominin* editing amino acid distributions for the NFS1 and SFS fly populations. The proportion of the different *prominin* editing isoforms for NFS1 and SFS fly lines, using editing levels from the 25˚C mmPCR-seq data, and ordered from lowest to highest editing of site 770. Editing levels for each fly line represent the average of two single flies (biological replicates). "NY" (red) represents both site 770 and site 773 unedited, "NC" (brown) represents site 770 unedited and site 773 edited, "SY" (turquoise) represents site 770 edited and site 773 unedited, and "SC" (purple) represents both site 770 and site 773 edited.

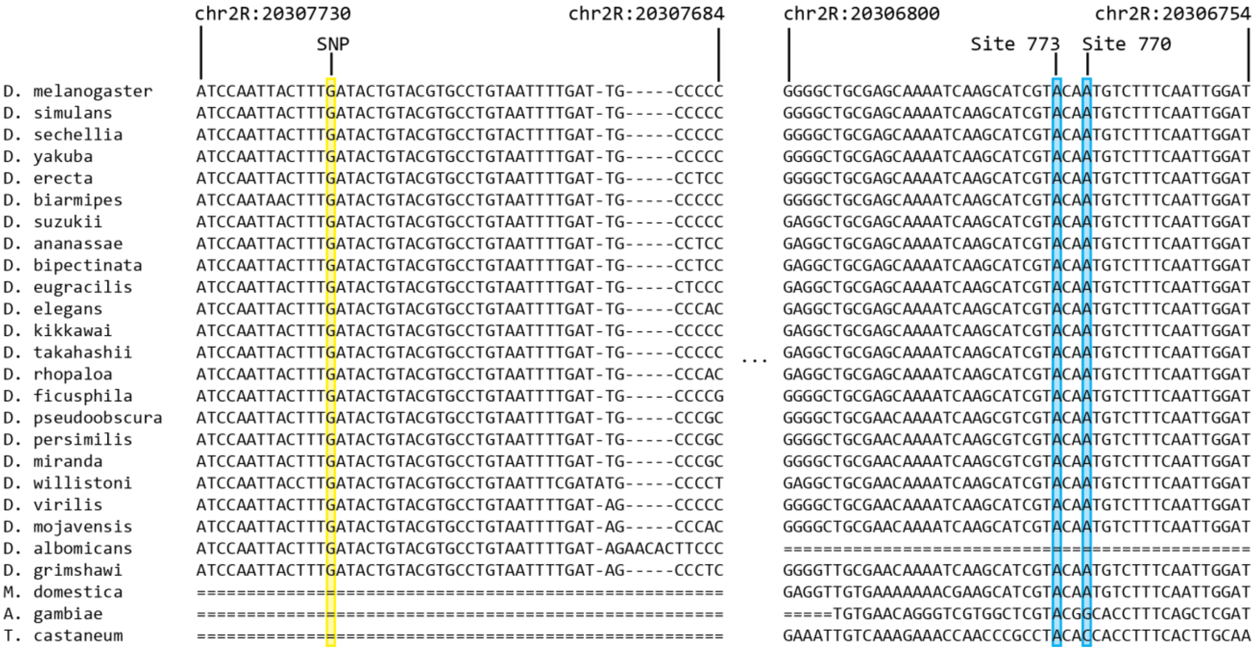

Supplementary Figure 15: Conservation of the predicted *prominin* ECS and editing stem region. Comparing the sequences of the predicted *prominin* ECS and editing stem region between 26 *Drosophila* and related insect species. The SNP that is correlated with *prominin* editing levels is highlighted in yellow, while the two editing sites are highlighted in blue.

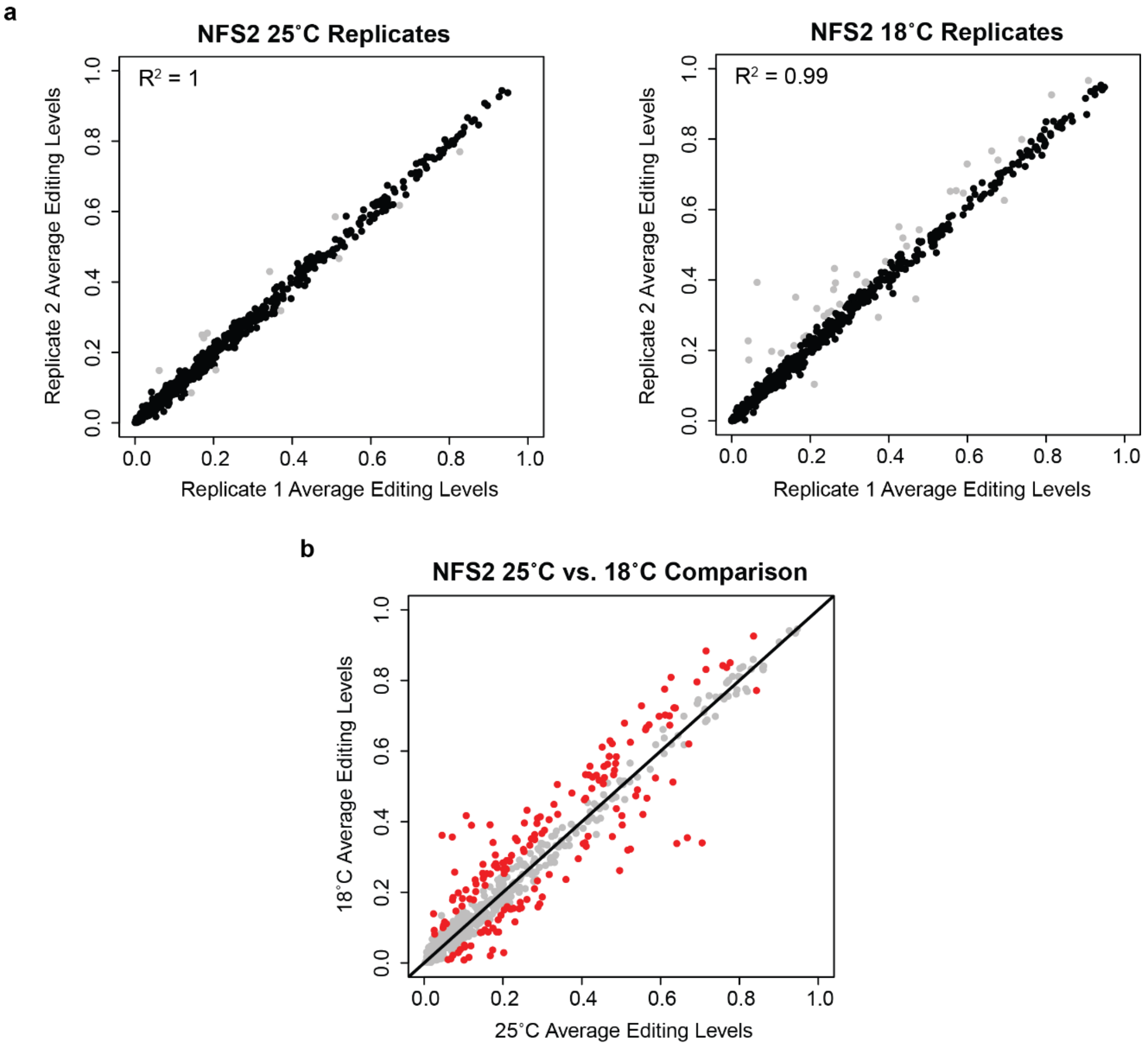

Supplementary Figure 16: Temperature regulation of RNA editing in the NFS2 fly population. (a) Scatterplots comparing average biological replicate 1 and replicate 2 mmPCR-seq editing levels for NFS2 flies at 25°C and at 18°C. Gray points represent sites with > 5% editing level differences between replicates. (b) Scatterplot comparing the average editing levels of NFS2 flies between 25°C and 18°C. Red dots represent sites with > 5% editing level difference between the temperatures, and FDR-adjusted p-value < 0.05 (t-test). The number of fly lines represented for each of these sites per population ranges from 3 to 5 for NFS2 and 3 to 16 for SFS; see Supplementary Data 8.

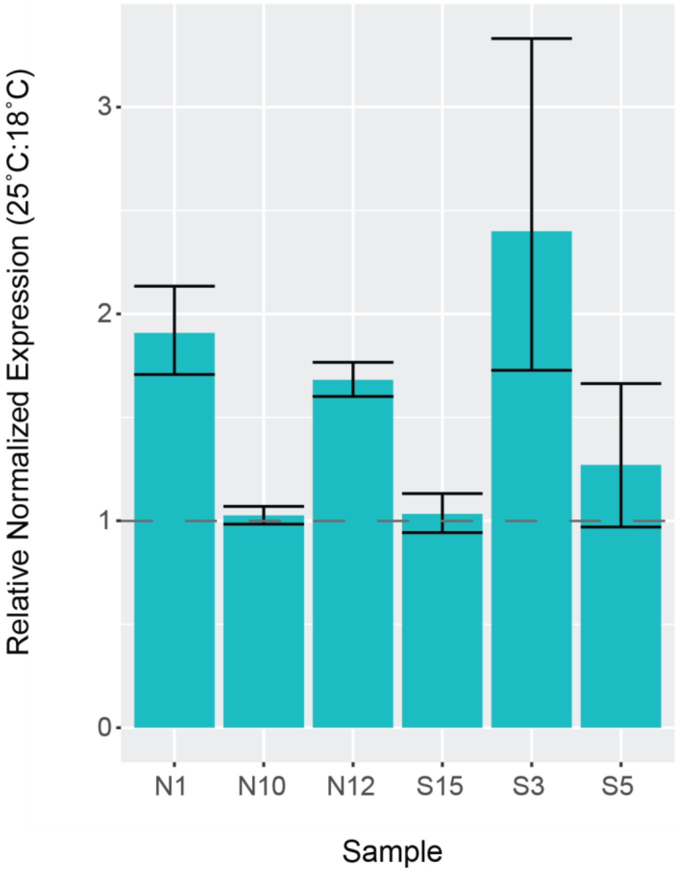

Supplementary Figure 17: Temperature regulation of *Adar* gene expression. Scatterplot comparing average *Adar* gene expression at 25˚C versus 18˚C for six Evolution Canyon lines. Error bars represent standard error of the mean of the two biological replicates tested per sample and per temperature condition.

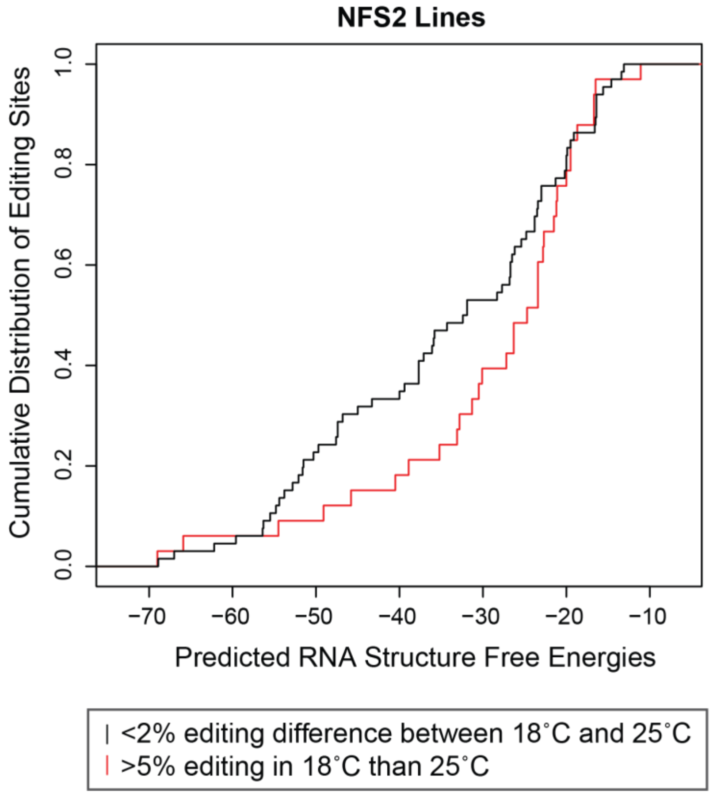

Supplementary Figure 18: Predicted RNA structure free energies for sites affected and not affected by temperature in the NFS2 fly population. For NFS2 flies, plot comparing predicted RNA structure free energy levels for sites that show < 2% editing level difference between 18°C and 25°C (black), and sites that show > 5% editing at 18°C than 25°C (red) (p-value = 0.054, one-sided KS-test).

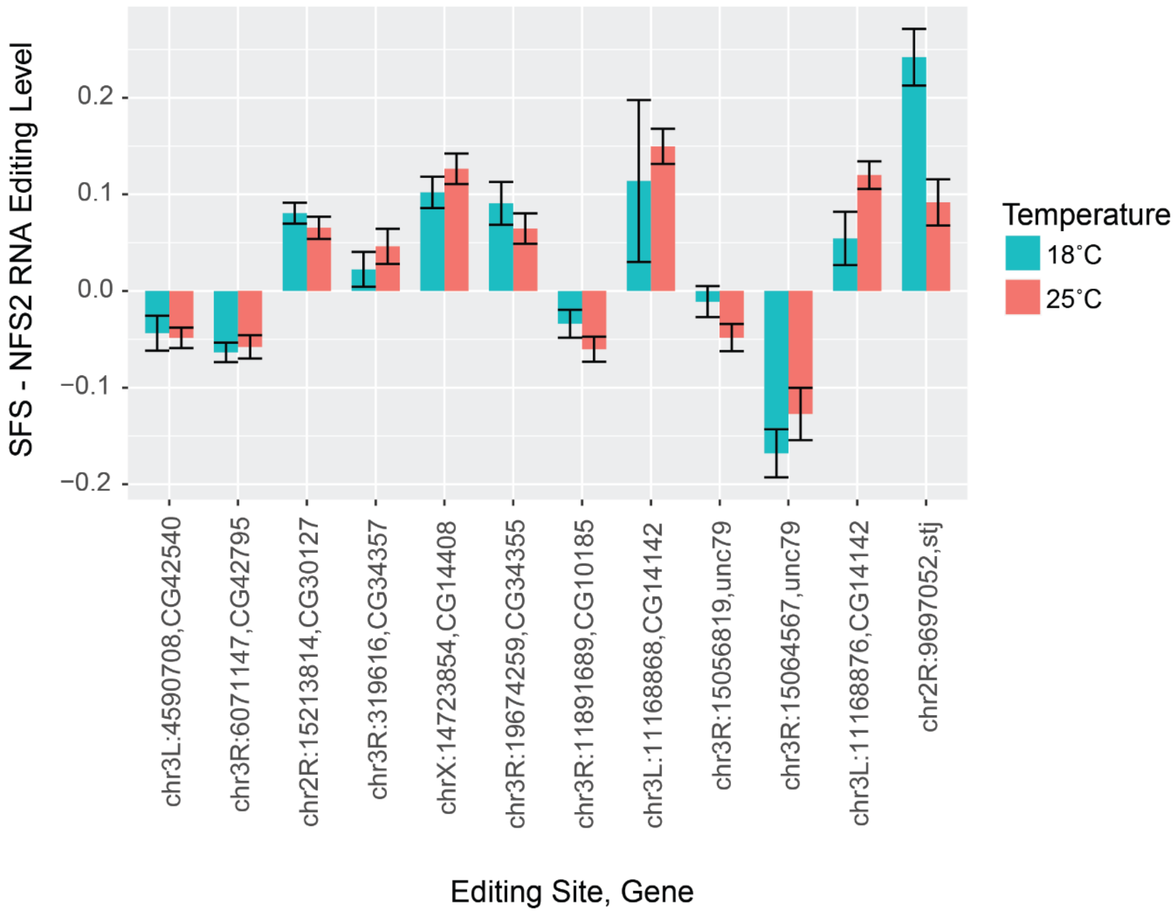

Supplementary Figure 19: Comparing genetic and temperature regulation of RNA editing in the NFS2 and SFS fly populations. For the differentially edited mmPCR-seq sites shown in Supplementary Figure 11 that had adequate coverage for samples at 18°C (see Methods for details), comparing average editing level differences between the SFS and NFS2 fly populations at 18°C (turquoise) and 25°C (red). Error bars represent standard deviation. The number of fly lines represented for each of these sites per population ranges from 3 to 5 for NFS2 and 15 to 16 for SFS; see Supplementary Data 9.

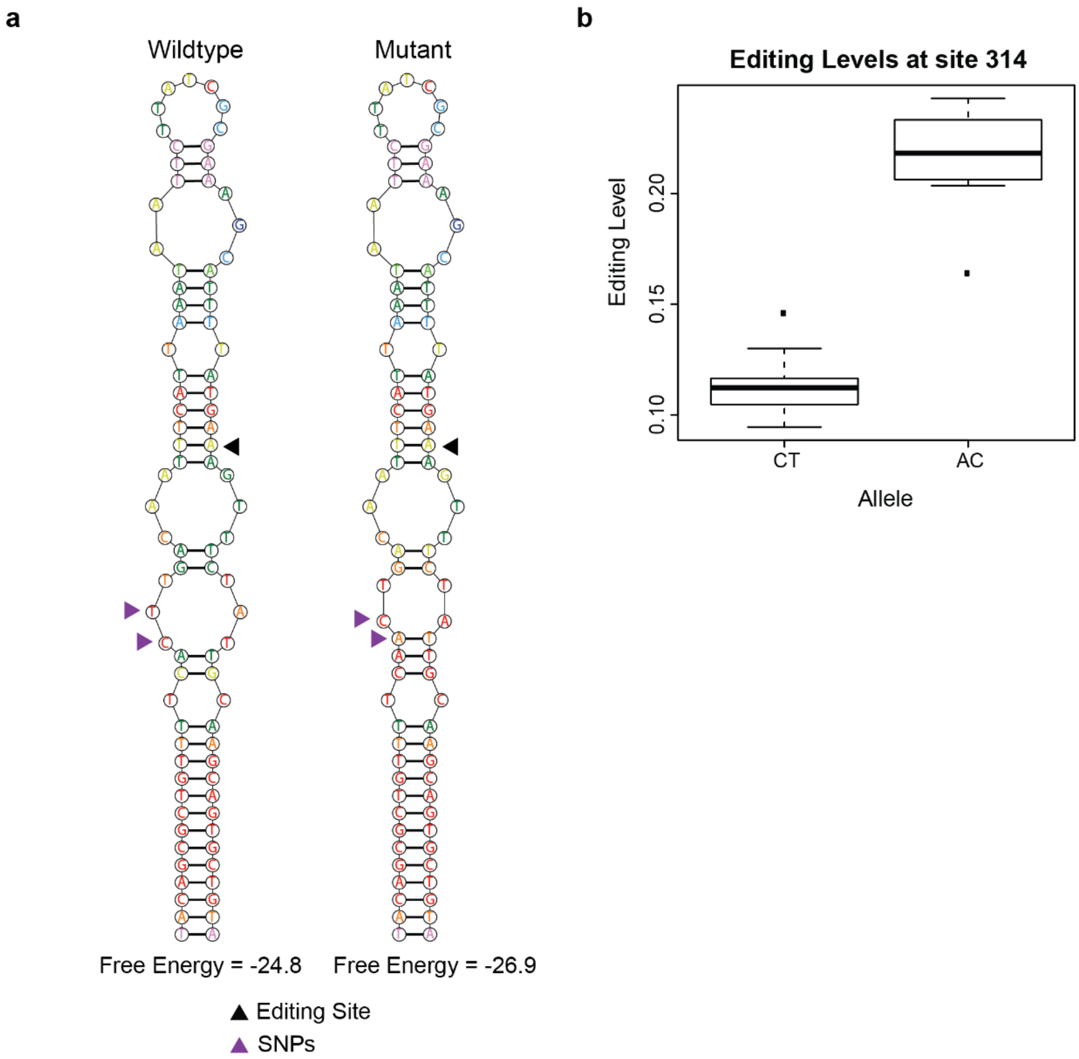

Supplementary Figure 20: Two SNPs in the ECS of *falafel* are associated with its editing levels. (a) RNA structure predictions and corresponding free energies for *falafel* editing site 314 (chr3R:9518314) and its corresponding ECS, for both wildtype *falafel* and with SNPs in the *falafel* ECS that are correlated with the site's editing levels. (b) Boxplot showing editing levels for *falafel* site 314 for 8 Evolution Canyon fly lines with wildtype alleles and 24 Evolution Canyon fly lines with mutant alleles.

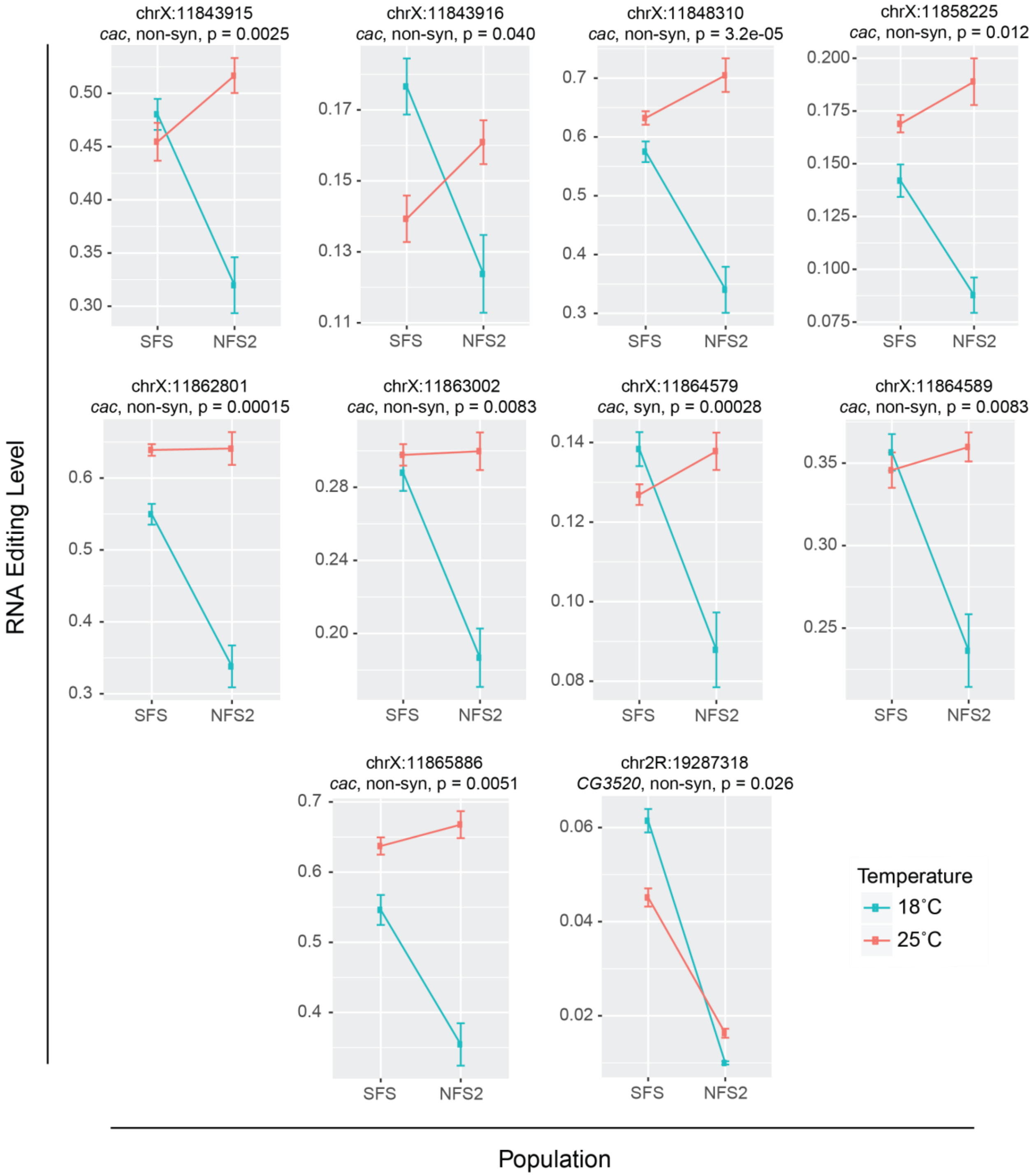

Supplementary Figure 21: Editing sites with significant interactions between genetics and environment in the NFS2 and SFS fly populations. Plots showing editing levels of sites that display a significant interaction (adjusted p-value < 0.05) between genetics and environment. Editing levels at 18°C are shown in turquoise and at 25°C are shown in red. The gene containing the editing site, annotation of the editing site, and p-values are listed for each site (p-values represent FDR-adjusted p-values, ANOVA test). Error bars represent standard error of the mean. The number of fly lines represented for each of these sites per population is 5 for NFS2 and 14 to 16 for SFS; see Supplementary Data 10.
