# Supplementary material for: Regulation of gene expression and RNA editing in Drosophila adapting to divergent microclimates
Source: Nat Commun. 2017 Nov 17;8:1570. doi: 10.1038/s41467-017-01658-2 (PMC5691062; doi:10.1038/s41467-017-01658-2)
Supplement: Supplementary file 3 — Description of Additional Supplementary Files [file 41467_2017_1658_MOESM3_ESM.pdf]

File Name: Supplementary Data 1
Description: Locations of selective sweeps in the NFS1, NFS2, and SFS fly populations from Evolution Canyon.

File Name: Supplementary Data 2
Description: Significantly differentially expressed genes between the NFS1 and SFS and between the NFS2 and SFS fly populations in head and whole body tissue.

File Name: Supplementary Data 3
Description: SNP enrichment in the genes and promoter regions (1kb upstream of transcription start site) of significantly differentially expressed genes between the NFS2 and SFS fly populations.

File Name: Supplementary Data 4
Description: Top 0.5% Fst SNPs between the NFS1 and SFS and between the NFS2 and SFS fly populations that also occur in selective sweeps in those respective populations.

File Name: Supplementary Data 5
Description: Top 0.5% Fst SNPs that occur in the genes or promoter regions (1kb upstream of transcription start site) of significantly differentially expressed genes between the NFS1 and SFS and between the NFS2 and SFS fly populations, as well as in selective sweeps in those respective populations.

File Name: Supplementary Data 6
Description: Significantly differentially edited sites (FDR-adjusted p-value < 0.05) between the NFS1 and SFS populations and between the NFS2 and SFS fly populations, identified through mmPCR-seq.

File Name: Supplementary Data 7
Description: Significantly differentially edited sites (FDR-adjusted p-value < 0.05) between the NFS1 and SFS populations and between the NFS2 and SFS fly populations, identified through RNA-seq of head tissue.

File Name: Supplementary Data 8
Description: Significantly differentially edited sites (FDR-adjusted p-value < 0.05 and editing level difference > 5%) between flies raised at 18˚C and 25˚C in the NFS1, NFS2, and SFS fly populations.

File Name: Supplementary Data 9
Description: Editing level differences between NFS1 and SFS and between NF2 and SFS at both 18˚C and 25˚C, for significantly differentially edited sites (FDR-adjusted p-value < 0.05 and editing level difference ≥ 5%) between those respective populations at 25˚C.

File Name: Supplementary Data 10

Description: Editing sites that show significant interactions (FDR-adjusted p-value < 0.05) between genetics and environment in the NFS2 and SFS fly populations.
